# Can measurement of glucose levels in blood and peritoneal fluid be used to diagnose septic peritonitis in dogs?

**DOI:** 10.18849/ve.v7i1.382

**Published:** 2022-03-02

**Authors:** Nicole D'Mello+

**Keywords:** ABDOMINAL EFFUSION, CANINE, DIAGNOSIS, DOGS, GLUCOSE, SEPTIC PERITONITIS

## Abstract

PICO question

Can the measurement of blood and peritoneal fluid effusion glucose levels be used to accurately diagnose septic peritonitis in dogs when compared to cytology and bacterial culture?

Clinical bottom line

Category of research question

Diagnosis

The number and type of study designs reviewed

Three papers were critically reviewed, all of which were diagnostic test evaluation studies

Strength of evidence

Moderate

Outcomes reported

Glucose measurements can be used to diagnose septic peritonitis when the blood plasma glucose level is >2.1 mmol/L higher than that of the peritoneal fluid glucose when using a veterinary point of care (POC) glucometer. If using a biochemistry analyser, a whole blood glucose >1.1 mmol/L higher than that of the peritoneal fluid can be used to diagnose septic peritonitis. This is only relevant when the peritoneal fluid is collected by abdominocentesis and not in a postoperative period

Conclusion

At present, there is moderate evidence that glucose measurements are useful as a patient side test for the diagnosis of septic peritonitis and are especially useful in cases where intracellular bacteria cannot be identified on cytology. However, despite the so far promising accuracy results, the cut-offs reported are quite variable and overall, there is not a single diagnostic test that is 100% sensitive and specific in repeated studies. Therefore, the results of the glucose measurements should be evaluated alongside other biomarker testing, imaging modalities and the clinical presentation of the patient. Glucose measurements cannot currently replace culture / sensitivity and cytology as the gold standard for the diagnosis of septic peritonitis

## How to apply this evidence in practice

The application of evidence into practice should take into account multiple factors, not limited to: individual clinical expertise, patient’s circumstances and owners’ values, country, location or clinic where you work, the individual case in front of you, the availability of therapies and resources.

Knowledge Summaries are a resource to help reinforce or inform decision making. They do not override the responsibility or judgement of the practitioner to do what is best for the animal in their care.

## Clinical scenario

A veterinarian is working the out of hours shift when a client brings in their 5 year old Golden Retriever. The dog is a known scavenger and is now presenting with anorexia, lethargy and abdominal pain. A quick ultrasound scan shows a small amount of free fluid in the abdomen. The veterinarian suspects this is case of septic peritonitis but they could not find any intracellular bacteria in the peritoneal fluid sample they took and cannot wait for results from bacterial culture. The veterinarian wonders if they can measure the glucose levels in blood and peritoneal fluid instead to reliably confirm their working diagnosis.

## The evidence

The search identified three diagnostic test evaluation studies that were related to the PICO question. The experimental designs provide a moderate strength of evidence as the studies were generally well planned, the patient follow-up was good and all studies were carried out in a realistic clinical setting. The main limitation in all the studies was the small sample size.

## Summary of the evidence

**Bonczynski et al. (2003):** 

Population:	Mixed population of dogs and cats with naturally occurring peritoneal effusions, presenting at a referral practice.
Sample size:	18 dogs: Seven septic peritoneal effusions 11 nonseptic peritoneal effusions (control group)
Intervention details:	Minimum of 1 ml of venous blood and 1 ml of peritoneal effusion collected from all patients and placed into heparinised tubes. The samples were frozen immediately and analysed by the same person each time within 24 hours.
Study design:	Diagnostic test validation study.
Outcome studied:	All objective assessments (relevant to the PICO question): Glucose was measured on all blood and peritoneal fluid samples using a blood chemistry analyser; VetTest 8008 analyser (VetTest Analyzer; Idexx). Total nucleated cell count was measured on peritoneal fluid in all patients. The blood to fluid glucose (BFG) were measured by subtracting the peritoneal fluid concentration from the blood concentration. All peritoneal fluid samples were submitted for cytology (for presence of intracellular bacteria) and bacterial culture to determine if patients had septic peritonitis (SP) or nonseptic peritonitis (NSP). Sensitivity, specificity, positive predictive value (PPV) and negative predictive value (NPV) were determined. Other biomarkers were measured in this study however they were not directly relevant to the PICO question and therefore will not be commented on further in this Knowledge Summary.
Main Findings (relevant to PICO question)	Peritoneal fluid glucose concentration was significantly lower (P = 0.008) in dogs with septic effusion than those with nonseptic effusion. The median BFG difference was significantly higher in dogs with septic effusion than those with nonseptic effusion (P = 0.0005). A BFG difference >20 mg/dL (>1.1 mmol/L) was 100% sensitive and specific for diagnosis of SP in dogs, with PPV and NPV of 100%. In septic dogs, peritoneal fluid glucose measurements <50 mg/dL (<2.8 mmol/L) were 57% sensitive and 100 % specific, with a PPV and NPV of 83%. BFG difference is more reliable for diagnosis of SP than peritoneal fluid glucose measurements alone.
Limitations:	Small sample size of 18 dogs reduces the reliability of the results. There is an absence of descriptive statistics (breed, age, gender, etc.) for the study population. This is relevant as a more varied population has a greater applicability to general clinical practice. The glucose concentration was determined using a blood chemistry analyser which is not always practical for emergency patient side testing. The control population of dogs were not matched based on clinical history, exam findings or severity of illness and were not that clinically similar to dogs with SP. Not all the controls had an inflammatory effusion, some were neoplastic. Animals were sampled only once (at presentation). This does not allow for mapping of the changes in biomarker concentration over time. Time since onset of disease may affect biomarker concentrations and therefore may reduce the reliability and significance of the results. This study was conducted in a referral practice. Patients presenting are typically at a severe stage in the disease process. Results from this study may not be directly applicable to patients with the same disease at an earlier or milder stage in the disease process, as is often seen in first opinion general practice.

**Koenig & Verlander (2015):** 

Population:	Mixed population of dogs with naturally occurring peritoneal effusions presenting at referral hospitals.
Sample size:	39 dogs: 17 septic peritonitis (SP); Of which six were receiving antibiotics at the time of sampling; 22 nonseptic peritonitis (NSP) (control group); Of which 10 were receiving antibiotics at the time of sampling.
Intervention details:	All samples were collected concurrently on first presentation of peritoneal effusion: Collection of venous whole blood, kept in heparinised tube. Collection of peritoneal fluid via aseptic abdominocentesis. All samples were analysed with a veterinary point of care (POC) glucometer immediately after collection. Samples were then centrifuged to obtain plasma and peritoneal supernatant and then tested with a veterinary POC glucometer.
Study design:	Diagnostic test validation study.
Outcome studied:	Objective outcomes studied (relevant to PICO question): Glucose concentration in whole blood and peritoneal fluid using a veterinary POC glucometer. Glucose concentration in plasma and peritoneal fluid supernatant using a veterinary POC glucometer. Peritoneal fluid cytology for bacterial identification and bacterial culture for diagnosis of SP. Sensitivity, specificity, positive predictive value (PPV) and negative predictive value (NPV) of glucose concentration differences for whole blood to peritoneal fluid (WB-PF), plasma to peritoneal fluid (P-PF) and plasma to peritoneal fluid supernatant (P-PFS) for the diagnosis of SP was determined. Other biomarkers were measured in this study however they were not directly relevant to the PICO question and therefore will not be commented on further in this Knowledge Summary.
Main Findings (relevant to PICO question)	There was a significant and clinically relevant difference in glucose concentration when measured with a veterinary POC glucometer in whole blood versus plasma (P < 0.001) but not between peritoneal fluid and peritoneal supernatant (P = 0.140). When using a veterinary POC glucometer with a cut off of >20 mg/dL (>1.1 mmol/L): WB-PF difference was not an acceptable method of diagnosing SP with a sensitivity of 41.2%, specificity of 100%, PPV of 100% and NPV of 68.8%. P-PF difference improved the diagnostic accuracy with sensitivity of 88.2%, specificity of 80%, PPV of 78.9% and NPV of 88.9%. When using a veterinary POC glucometer with a cut-off of >38 mg/dL (>2.1 mmol/L): P-PF difference improved the diagnostic accuracy with sensitivity of 88.2%, specificity of 100%, PPV of 100% and NPV of 90.9%. P-PF difference had the best diagnostic accuracy at 94.6%.
Limitations:	One dog included in the study was administered dextrose before blood glucose was measured which can elevate blood glucose measurements therefore causing a greater difference between blood and peritoneal fluid glucose concentrations. It may have been assumed that this difference was due to the disease process rather than the administration of dextrose. For a diagnosis of SP, patients had to have bacteria identified on cytology or a positive culture result. However, only 3/22 dogs with nonseptic peritoneal effusion had peritoneal fluid sent for culture. This could have led to a misdiagnosis of a sterile effusion based on a combination of results from cytology and post mortem findings but without the added negative culture result. This may have affected the sensitivity and specificity of the study. If the glucose level in any sample was too low to give an objective reading, the patient would be assigned a glucose level of 20 mg/dL (1.1 mmol/L). In one case this meant there was a minimal difference in fluid glucose as the patients’ blood glucose was 28 mg/dL (1.55 mmol/L) and so the cut-off value of >20 mg/dL could not be reached. Therefore, the patient would have been misdiagnosed with a nonseptic effusion. This reduces the sensitivity of the data in terms of using a veterinary POC glucometer and may explain why using a biochemistry analyser is more sensitive. The small sample size reduces the reliability of the results. The researcher conducting the fluid analysis was not blinded to the patient presentation, clinical signs or any other test results. Researcher bias will affect the validity of the result, although in this case it may be less significant as the fluid analysis is an objective measure. This study was conducted in a referral practice. Patients presenting are typically at a severe stage in the disease process. Results from this study may not be directly applicable to patients with the same disease at an earlier or milder stage in the disease process, as is often seen in first opinion general practice.

**Martiny & Goggs (2019):** 

Population:	Mixed population of dogs with naturally occurring systemic inflammatory response syndrome and / or peritoneal effusion, presenting at primary and referral practice.
Sample size:	37 dogs: 18 with septic peritonitis (SP); Diagnosed by either positive bacterial culture, intracellular bacteria on cytology or gross evidence of gastrointestinal perforation at surgery. 19 with nonseptic ascites (NSA) (age-matched controls).
Intervention details:	Peritoneal fluid sampled via abdominocentesis and placed into heparinised, ethylenediaminetetraacetic acid (EDTA), 2% sodium citrate and plain tubes. Blood samples were collected via venipuncture or intravenous catheterisation and placed into heparinised, EDTA, 3.2% sodium citrate and plain tubes.
Study design:	Diagnostic test evaluation study.
Outcome studied:	Samples from SP (18) were paired with (19) age-matched controls for statistical analysis. Objective assessments: After sample collection, glucose measurements were immediately obtained using a RapidPoint 405 (Siemens Medical Solutions, Norwood), on all heparinised blood and effusion samples (not supernatant).
Main Findings (relevant to PICO question)	Blood-effusion glucose gradient of <37 mg/dL (<2.06 mmol/L) was 89.5% sensitive for the diagnosis of SP but had a low specificity of 66.7%. Blood-effusion gradient of glucose in SP dogs had a mean of 3.3 mmol/L and in NSA dogs a mean of 0.3 mmol/L which was a statistically significant difference. Blood-effusion gradient of glucose had an area under the receiver operating characteristics (AUROC) value of 0.809 which was significant (P = 0.0013) when compared to the line of identity AUROC value of 0.5. No single biomarker is completely discriminating for SP and no cut-off offers 100% sensitivity and specificity.
Limitations:	Animals were sampled only once however the biomarker concentrations can vary as the disease progresses. This can affect the validity of the results. The small sample size reduces the reliability of the results. The personnel carrying out cytological evaluation of peritoneal fluid varied in their levels of training and experience which could lead to either false negative or false positives in the diagnosis of SP or NSA which would affect the reliability of the results. A standardised approach ensuring people with the same level of training in cytological evaluation carried out the analysis, would achieve more reliable results. The researcher conducting the fluid analysis was not blinded to patient presentation, clinical signs and other test results, although the effects of not blinding will have less of an impact when using objective measurements. This study was conducted in a referral practice. Patients presenting are typically at a severe stage in the disease process. Results from this study may not be directly applicable to patients with the same disease at an earlier or milder stage in the disease process, as is often seen in first opinion general practice.

## Appraisal, application and reflection

The literature search revealed three papers that were directly relevant to the PICO question outlined. These were three diagnostic test evaluation studies (Bonczynski et al., 2003; Koenig & Verlander, 2015; and Martiny & Goggs, 2019).

There were two prospective clinical trials (Szabo et al., 2011; and Guieu et al., 2016) that analysed blood and peritoneal fluid samples following the placement of closed-suction abdominal drains (CSAD) after surgery, rather than evaluating patients in an acute presentation. Although glucose measurements were analysed, both studies did not identify the sensitivity, specificity, positive predictive value (PPV), negative predictive value (NPV) or diagnostic accuracy of any of the tests used and so the studies were not included for summary in this report. However, the outcomes highlighted from these studies were that glucose measurements were not reliable in diagnosing septic peritonitis (SP) in postoperative cases in which the peritoneal fluid sampled was collected from a closed suction drain. The study by Szabo et al. (2011) used an experimental design in which healthy patients underwent abdominal surgery and were then monitored, which reduces the validity of the results to be applied to clinical practice. The results from these studies are specific to these clinical scenarios and the data should not be applied to patients presenting in an acute scenario or with fluid collected by abdominocentesis.

The diagnostic test evaluation studies (Bonczynski et al., 2003; Koenig & Verlander, 2015; and Martiny & Goggs, 2019) all provided a moderate level of evidence. The studies admitted patients to the study group consecutively over a time period and in accordance to pre-defined inclusion criteria which minimises selection bias. In terms of patients selection, Bonczynski et al. (2003) included a wider range of inclusion criteria which meant that all patients with effusions (inflammatory or neoplastic) were admitted to the study group, which may be more representative of cases seen in practice. This study identified a diagnostic sensitivity and specificity of 100% when using blood to fluid glucose (BFG) differences of >1.1 mmol/L to diagnose SP (Bonczynski et al., 2003). However, Martiny & Goggs (2019) identified that BFG gradients did not have a sensitivity, specificity or diagnostic accuracy of 100%. This was likely due to the patient selection for this study. All patients had to have an effusion as well as systemic inflammatory response syndrome (SIRS) diagnosed at presentation. This meant that the SP group and the control group of nonseptic ascites (NSA) were clinically very similar and were also age-matched. This study identified that blood-effusion gradients were higher in dogs with SP compared to NSA and this was a statistically significant difference. They also identified that blood-effusion gradients had an area under the receiver operating characteristics (AUROC) value of 0.809 which was significant (p-value of 0.0013) when compared to the line of identity AUROC value of 0.5, which means that blood-effusion gradients are able to discriminate between SP and NSA (Martiny & Goggs, 2019). However, the study did not identify a cut-off value for blood-effusion glucose gradients with reasonable sensitivity or specificity, although they did identify that a cut-off of <2.06 mmol/L had a poor specificity of 66.7% but reasonable sensitivity of 89.5%.

It is important to note that the studies by Bonczynski et al. (2003) and Martiny & Goggs (2019) both analysed glucose measurements using blood chemistry analysers and blood gas analysers respectively. This can limit the application of the findings in each study to clinical practice as these diagnostic tools are not always readily available. The study by Koenig & Verlander (2015) overcame this by measuring glucose using a veterinary point of care (POC) glucometer, a tool commonly available in veterinary practice. The study identified that whole blood to peritoneal fluid (WB-PF) concentration differences were insensitive for diagnosis of SP as was plasma to peritoneal fluid, if a cut-off value of >1.1 mmol/L was used. However, this study did identify that a plasma to peritoneal fluid (P-PF) glucose concentration difference greater than >2.1 mmol/L (>38 mg/dL) supported an accurate diagnosis of SP in dogs with peritoneal effusions, with a diagnostic sensitivity of 88.2%, specificity of 100%, PPV of 100% and NPV of 90.9% giving a diagnostic accuracy of 94.6% (Koenig & Verlander, 2015). It is also important to note that when using a veterinary POC glucometer, plasma rather than whole blood produced significantly relevant differences (P <0.001). However, there was no relevant difference when using peritoneal fluid rather than peritoneal fluid supernatant (P = 0.140) (Koenig & Verlander, 2015).

All of the diagnostic test evaluation studies (Bonczynski et al., 2003; Koenig & Verlander, 2015; and Martiny & Goggs, 2019) carried out a variety of diagnostic tests, including the gold standard reference tests of either cytology and culture and sensitivity, on all patients. This meant that every case of SP was confirmed by cytology and bacterial culture, so the accuracy of the index diagnostic test (blood and peritoneal fluid glucose measurements) was less likely to be overestimated. Each study also contained sufficient information for replicability if needed. However, in the studies by Koenig & Verlander (2015) and Martiny & Goggs (2019), the researcher carrying out the fluid analysis was not always blinded to the clinical signs, presentation or results of the other diagnostic test. This can lead to an element of conscious bias due to lack of blind independent interpretation, although the significance of this is less important when the diagnostic tests are measuring an objective variable such as glucose concentrations, as in this case. The main limitation for all the studies analysed in this paper is the small sample size which can result in larger confidence intervals, decreasing the power of the study and potentially, its clinical relevance. It is also important to note that all of the studies (Bonczynski et al., 2003; Koenig & Verlander, 2015; and Martiny & Goggs, 2019) collected data from patients in referral practice. It can be argued that these patients may be presenting with a more severe disease status than those seen in first opinion practice, therefore not being representative of all patients presenting at varying stages of disease. This can make it difficult to make direct applications from the data from these studies to first opinion general practice. Furthermore, it is important to note that all the studies (Bonczynski et al., 2003; Koenig & Verlander, 2015; and Martiny & Goggs, 2019) only sampled the animals once, typically at the initial presentation. Single time point sampling does not allow for identification of any changes in biomarker concentration over time therefore, the effect of disease progression on biomarker concentrations cannot be determined, limiting the reliability of these results.

The current gold standard for the diagnosis of SP in the pre-operative patient is either the identification of intracellular bacteria on cytology of peritoneal fluid or a combination of a positive bacterial culture of peritoneal fluid alongside cytology results confirming an inflammatory peritoneal effusion. Cytology for diagnosis of SP has a reported accuracy of between 57% (Mueller et al., 2001) to 87% (Lanz et al., 2001) with bacterial culture results yielding an accuracy of between 50% (Lanz et al., 2001) to 83% (Mueller at al., 2001). The wide range in diagnostic accuracy shows a clear limitation to the gold standard. Furthermore, cytological evaluation is subjective and dependent on the skill and expertise of the person analysing the sample. Results from bacterial cultures of peritoneal fluid will require around 2–3 days to yield bacterial growth. This is a clear limitation for the critical patient in which decisions must be made in a timely manner to improve patient outcomes. Therefore, an accurate patient side diagnostic test would be useful in aiding the diagnosis of SP.

In summary, three papers were identified in the literature search, providing a moderate level of strength of evidence. The research has highlighted that a P-PF glucose difference of >2.1 mmol/L (>38 mg/dL) can be used to aid in diagnosis of SP, with a sensitivity of 88.2%, specificity of 100% and diagnostic accuracy of 94.6%, when the peritoneal fluid has been acquired directly from abdominocentesis during the pre-operative period and if measured using a veterinary POC glucometer (Koenig & Verlander, 2013). However, when using a biochemistry analyser the difference can be lowered to 1.1 mmol/L (>20 mg/dL) with 100% specificity and sensitivity (Bonczynski et al., 2003). Throughout the research presented there is a large variety in suggested cut-off values when measuring glucose differences. This is due to different studies using biochemistry analysers rather than veterinary POC glucometers as well as comparing whole blood versus plasma and peritoneal fluid versus peritoneal supernatant. This makes it challenging to easily generalise the results to clinical practice. A further study could be carried out to directly compare the sensitivity, specificity and diagnostic accuracy when using different analysers and sample types which may make the results easier to summarise and apply to general practice.

Based on the strength of the studies analysed, there is only moderate evidence that glucose measurements are useful as a patient side diagnostic test for septic peritonitis therefore this should not be the sole diagnostic test carried out. Instead, glucose measurements should be used to build the clinical picture, alongside the patients clinical presentation, information gathered from imaging modalities and any results from cytology and / or bacterial culture of peritoneal fluid.

## Methodology Section

**Search Strategy:** 

Databases searched and dates covered:	CAB Abstracts on OVID Platform (2000–2021) PubMed NCBI (2000–2021)
Search strategy:	CAB Abstracts: (dogs or dog or canine or canines) AND (septic) AND (peritonitis or effusion) AND (glucose) PubMed: (dogs or dog or canine or canines) AND (septic) AND (peritonitis or effusion) AND (glucose)
Dates searches performed:	17 Oct 2021

**Exclusion / Inclusion Criteria:** 

Exclusion:	Papers not written in English. Papers not measuring blood and peritoneal fluid glucose or not diagnosing septic peritonitis via the gold standard of culture and sensitivity. This includes any papers not identified as a diagnostic test evaluation study in which diagnostic sensitivity and specificity was not identified (not relevant to PICO question). Review articles or conference proceedings. Papers with no author name, abstract or DOI number.
Inclusion:	Papers that measured glucose in blood and peritoneal fluid as well as confirmed the diagnosis of septic peritonitis via cytology and / or bacterial culture. The paper must determine diagnostic sensitivity and specificity for using glucose measurements to diagnose septic peritonitis.

**Search Outcome:** 

Database	Number of results	Excluded – Not written in English	Excluded – Not relevant to PICO question	Excluded – Review articles or conference proceedings	Excluded – No author name, abstract or DOI	Total relevant papers
CAB Abstracts	17	4	9	1	0	3
PubMed	10	0	7	0	0	3
Total relevant papers when duplicates removed						3

## Conflict of interest

The authors declare no conflicts of interest.

## Disclaimer

Knowledge Summaries are a peer-reviewed article type which aims to answer a clinical question based on the best available current evidence. It does not override the responsibility of the practitioner. Informed decisions should be made by considering such factors as individual clinical expertise and judgement along with patient’s circumstances and owners’ values. Knowledge Summaries are a resource to help inform and any opinions expressed within the Knowledge Summaries are the author’s own and do not necessarily reflect the view of the RCVS Knowledge. Authors are responsible for the accuracy of the content. While the Editor and Publisher believe that all content herein are in accord with current recommendations and practice at the time of publication, they accept no legal responsibility for any errors or omissions, and make no warranty, express or implied, with respect to material contained within. For further information please refer to our Terms of Use.
